# Orexin receptor antagonists as therapeutic agents for insomnia

**DOI:** 10.3389/fphar.2013.00163

**Published:** 2013-12-25

**Authors:** Ana C. Equihua, Alberto K. De La Herrán-Arita, Rene Drucker-Colin

**Affiliations:** ^1^Neuropatología Molecular, Instituto de Fisiología Celular, Universidad Nacional Autónoma de MéxicoMexico City, México; ^2^Center for Sleep Sciences and Medicine, Stanford UniversityPalo Alto, CA, USA

**Keywords:** orexin receptor antagonist, insomnia, hypocretins/orexins, therapy-related, sleep disorders

## Abstract

Insomnia is a common clinical condition characterized by difficulty initiating or maintaining sleep, or non-restorative sleep with impairment of daytime functioning. Currently, treatment for insomnia involves a combination of cognitive behavioral therapy (CBTi) and pharmacological therapy. Among pharmacological interventions, the most evidence exists for benzodiazepine (BZD) receptor agonist drugs (GABA_A_ receptor), although concerns persist regarding their safety and their limited efficacy. The use of these hypnotic medications must be carefully monitored for adverse effects. Orexin (hypocretin) neuropeptides have been shown to regulate transitions between wakefulness and sleep by promoting cholinergic/monoaminergic neural pathways. This has led to the development of a new class of pharmacological agents that antagonize the physiological effects of orexin. The development of these agents may lead to novel therapies for insomnia without the side effect profile of hypnotics (e.g., impaired cognition, disturbed arousal, and motor balance difficulties). However, antagonizing a system that regulates the sleep-wake cycle may create an entirely different side effect profile. In this review, we discuss the role of orexin and its receptors on the sleep-wake cycle and that of orexin antagonists in the treatment of insomnia.

## Introduction

Insomnia is the most common sleep disorder in the world. In the US alone, as much as 48% of the population reports experiencing transitory insomnia, while 22% suffers from insomnia almost every night as mentioned on the National Sleep Foundation website.

According to the second International Classification of Sleep Disorders (ICSD-2), insomnia is characterized by disturbed sleep that leads to impaired daytime functioning (e.g., fatigue, memory impairment, poor school performance, irritability, daytime sleepiness and proneness to errors, among other symptoms). Disturbed sleep can manifest as a difficulty in initiating/maintaining sleep, early morning awakening, or sleep that is chronically non-restorative or poor in quality, despite adequate opportunity for sleep to occur. Insomnia becomes a chronic problem when symptoms have been present for at least a month (NIH State-of-the-Science Conference Statement on Manifestations and Management of Chronic Insomnia in Adults, 2005).

The definition for insomnia disorder in the Diagnostic and Statistical Manual of Mental Disorders, 5th Edition, does not differ much from that of the ICSD-2 as it also includes complaints of dissatisfaction with sleep quantity or quality despite adequate opportunity to sleep and low performance in daytime functioning. In addition, the manual also includes a more specific timeframe where complaints occur at least three nights per week for at least 3 months.

The National Institutes of Health classifies insomnia as either primary (PI) or comorbid (previously referred to as secondary insomnia). PI refers to insomnia without comorbid conditions, whereas comorbid insomnia is employed when complaints arise in the context of another condition, such as depression, Parkinson's disease, rheumatoid arthritis, or restless leg syndrome; or as the side effect of a drug, such as caffeine, nicotine, alcohol or beta-blockers.

The etiology of PI is thought to be related to sustained physiological hyperarousal throughout the day. Management of insomnia can be achieved using cognitive behavioral therapy (CBTi) and/or pharmacological therapy. Common prescription medications for insomnia are benzodiazepine (BZD) receptor agonists (both BZDs and nonBZDs), sedating antidepressants and melatonin receptor agonists. Pharmaceutical intervention is often the first-line approach for the treatment of insomnia but still has many pitfalls, such as the development of tolerance, addiction and undesired side effects (including complex sleep related behaviors and abnormal thoughts).

Orexin (hypocretin) receptor antagonists are a new, promising pharmacological treatment for PI. The orexinergic system (otherwise known as the hypocretinergic system) has been strongly linked to the sleep/wake cycle (SWC) for its role in promoting and sustaining arousal (Piper et al., 2000; Xi et al., 2001). In addition, the antagonism of orexinergic receptors has been shown to induce somnolence in different species (Brisbare-Roch et al., 2007). In clinical trials orexin receptor antagonists have performed well, and subjects have reported improved quality of sleep with few side effects, the most common being complaints of mild headaches and dizziness.

In the first part of this review we discuss the current state of PI and the research that has led to the use of orexin receptor antagonists as therapy for PI. In the second part we focus on existing orexin receptor antagonists and their effectiveness in promoting sleep in animal models and managing insomnia in humans.

## Overview of the current treatment of insomnia

### CBTi

The main objective of CBTi is to tackle the cognitive and behavioral factors that could be perpetuating insomnia. The most frequent factors are excessive worrying about not sleeping enough and maladaptive behaviors such as spending excessive time in bed awake, excessive use of caffeine, and napping. Common techniques applied in CBTi include sleep hygiene education, cognitive restructuring, stimulus control, sleep restriction therapy and relaxation training (Morin et al., 1994; NIH State-of-the-Science Conference Statement on Manifestations and Management of Chronic Insomnia in Adults, 2005). Several studies have found that CBTi is an effective approach with long-term results for the treatment of insomnia (Morin, 1999; Jacobs et al., 2004).

The shortcomings of CBTi are related to access and adherence to treatment. Patients need to be trained by specialized medical practitioners, who are not readily available, and to stay highly motivated, as the therapy requires them to devote time to practicing and carrying out the techniques.

### Pharmacological treatments for insomnia

Pharmacological treatments for insomnia can broadly be classified as prescription FDA approved and non-prescription, over-the-counter (OTC), treatments.

#### Prescription FDA approved

Contemporary FDA approved pharmacological treatment includes GABA_A_ receptor agonists (BZDs and nonBZDs), sedating antidepressants and melatonin agonists. The use of a pharmacological therapy for the treatment of insomnia is somewhat easier than CBTi, but presupposes other difficulties, such as unresponsiveness to treatment, limited therapeutic potential, a poor side effect profile, tolerance and addiction. FDA medicines approved for the treatment of insomnia are listed in Table 1.

**Table 1:** **FDA approved medications for the treatment of insomnia**.

**Generic name**		**Therapeutic indication**	**Dosage (mg)**	**Known side effects**	**Mechanism of action**
**BENZODIAZEPINE RECEPTOR AGONISTS**					
BDZs	Estazolam	Insomnia	0.5–2	Dizziness, drowsiness, next day sedation, memory loss, anxiety, loss of coordination.	Positive allosteric modulator of GABA_A_ receptors
	Quazepam		7.5–15	Rebound insomnia.	
				Allergic reactions.	
	Triazolam		0.125–0.50	Complex sleep related behaviors: sleep-driving, making phone calls, eating.	
	Flurazepam		15–30	Abnormal thoughts and behavior: worsening of depression, suicidal thoughts or actions, increased aggressiveness.	
	Temazepam		7.5–30		
NonBDZs	Zaleplon	Sleep-onset insomnia	2.5–10	Dizziness, headache, drowsiness, nausea, vomiting.	
	Zolpidem	Sleep-onset and sleep maintenance insomnia	5–20	Complex sleep related behaviors.	
				Abnormal thoughts and behavior.	
	Eszopiclone		1–3	Physical dependence.	
**SEDATING ANTIDEPRESSANTS**					
Doxepin		Sleep-maintenance insomnia	3–6	Sedation, nasopharyngitis, gastrointestinal effects, hypertension.	5-HT & NE reuptake inhibitor H_1_ receptor antagonist
				Complex sleep related behaviors.	
				Abnormal thoughts and behavior.	
**MELATONIN RECEPTOR AGONISTS**					
Ramelteon		Sleep-onset insomnia	8	Drowsiness, tiredness, dizziness. Allergic reactions.	MT_1_ & MT_2_ receptor agonist
				Complex sleep related behaviors	
				Abnormal thoughts and behavior.	
				Hormone effects: decreased interest in sex, problems getting pregnant.	

All information was obtained from the FDA website (www.fda.gov). Abbreviations: BDZs, benzodiazepines; 5-HT, serotonin; NE, norepinephrine; H, histamine; MT, melatonin.

***BZDs and nonBZDs*.** The first FDA approved drugs for insomnia were BZDs (estazolam, quazepam, triazolam, flurazepam and temazepam) and nonBZDs, also known as z-drugs (zaleplon, zolpidem, and eszopiclone). These drugs, with the exception of eszopiclone, are effective for the short-term management of insomnia. Eszopiclone on the other hand, has been found to have sustained efficacy for up to 6 months (Table 1).

BZD and nonBZD compounds are GABA_A_ agonists. GABA_A_ receptors are pentameric receptors conformed of combinations of α (1–6), β (1–3), γ (1–3), δ (1), ε (1), π (1), and θ (1) subunits. The endogenous ligand GABA binds at the active site located at the interface of α- and β-subunits, instead, the binding site for BZDs and nonBZDs is located between α- and γ-subunits of α- and γ-subunit containing GABA_A_ receptors. Differences among BZDs and nonBZDs relate to their selectivity for different types of GABA_A_ receptors, while BZDs can bind to subunits of the α1, α2, α3, and α5 classes, nonBZDs preferentially bind to the α1 subclass (Rudolph and Knoflach, 2011).

Activation of GABA_A_ receptors tends to stabilize or hyperpolarize the resting potential, and can make it more difficult for excitatory neurotransmitters to depolarize the neuron and generate an action potential. The net effect is typically inhibitory, reducing the activity of the neuron. The GABA_A_ channel opens quickly and thus contributes to the early part of the inhibitory post-synaptic potential. This can lead to several undesired side effects that range from cognitive and psychomotor impairment, rebound insomnia, and anterograde amnesia, to increased risk of motor collisions and falls (Lader, 2012; Gunja, 2013).

***Sedating antidepressants*.** For a long time, antidepressants were used to treat insomnia in an off-label manner. Among these, the serotonin antagonist and reuptake inhibitor trazodone was the most popular. Then, in 2010, the FDA approved the tricyclic antidepressant (TCA) doxepin for the treatment of sleep maintenance insomnia (frequent nighttime or early morning awakenings).

There are different classes of antidepressants with sedating properties; in particular doxepin is classified as a serotonin and norepinephrine reuptake inhibitor TCA. Despite this, the sleep-promoting effects of doxepin are thought to relate mainly to its antihistaminergic properties (Risberg et al., 1975). In this regard, doxepin is a potent histamine H_1_ receptor antagonist (Richelson, 1979).

Therapeutic effects of doxepin are observed at very low dosages (3–6 mg/day), improving sleep maintenance without rebound insomnia or physical dependence. Common side effects include sedation, nasopharyngitis, gastrointestinal effects, and hypertension (Weber et al., 2010).

***Melatonin agonists*.** Melatonin is a natural hormone produced by the pineal gland following a circadian rhythm. The production of melatonin peaks when the lights go out, which signals the organism that it is nighttime (Reiter, 1986). In humans, melatonin has sleep-promoting effects as it has been found to induce sedation, lower core body temperature, reduce sleep latencies and increase total sleep time (Dollins et al., 1994; Zhdanova et al., 1996; Erman et al., 2006).

Ramelteon is an FDA approved melatonin agonist that acts upon MT_1_ and MT_2_ receptors improving sleep-onset latency at a recommended dose of 8 mg/day. The most common complaints users have described are headache, somnolence, dizziness and sore throat (Pandi-Perumal et al., 2011). Overall, ramelteon is very well tolerated, and unlike BZDs, residual effects such as cognitive and psychomotor impairments are absent (Johnson et al., 2006) (Table 1).

#### Non-prescription (OTC)

The most commonly used OTC sleep aids are antihistamines; other OCT include alcohol, valerian and l-tryptophan. Histaminergic neurons are mainly localized in the tuberomammillary nucleus (TMN) from where they project to many regions of the (CNS) system including the wake-promoting basal forebrain (BF) and orexinergic neurons in the lateral hypothalamus (LH) (Köhler et al., 1985; Panula et al., 1989). The sedating effects of antihistamines have been known for a long time (Risberg et al., 1975) and are thought to be related to inhibition of H_1_ receptor activity (Saitou et al., 1999).

Despite the popularity of OCT sedating antihistamines, these agents have several undesirable side effects that limit their usefulness as sleep aids (NIH State-of-the-Science Conference Statement on Manifestations and Management of Chronic Insomnia in Adults, 2005). In addition to antagonizing histamine receptors, these compounds often display anticholinergic effects (dry mouth, blurred vision, constipation, tachycardia, urinary retention, and memory deficits) and next-day impairment (Kay, 2000; Meoli et al., 2005).

### Neurobiological model of insomnia

PI, though classified as a sleep disorder, is thought to be a consequence of physiological hyperarousal during sleep and wakefulness. For example, objective sleepiness measures such as the Multiple Sleep Latency Test (MSLT), have failed to show increased sleepiness in insomniacs when compared to healthy controls (Edinger et al., 2003). Furthermore, insomniacs appear to be more alert following a night of poor sleep when compared to control subjects (Stepanski et al., 1988) and during sleep exhibit a surge of beta and gamma activity (Perlis et al., 2001), suggesting a generalized disorder that persists throughout the SWC. This model has been supported by studies that have detected physiological differences between insomniacs and controls. Monroe was the first to document that poor sleepers have increased physiological activity, which includes augmented heart rate, body temperature, oxygen consumption, secretion of cortisol, adrenocorticotropic hormone (ACTH) and adrenaline (Monroe, 1967; Adam et al., 1986; Vgontzas et al., 2001; Bonnet and Arand, 2010).

Elevated levels of free cortisol and ACTH in the urine are indicators of the overactivation of the hypothalamic-pituitary-adrenal axis (HPA) that could account for some of the symptoms of PI, including arousal, fragmented sleep and increased sleep latency (Steiger et al., 1991; Richardson and Roth, 2001).

The HPA plays a fundamental role in the stress response; increased levels of cortisol after a night of sleep loss have been interpreted as reflecting the stress of maintaining a state of vigilance (Chapotot et al., 2001). In normal conditions, cortisol levels somewhat parallel arousal throughout the day, reaching peak levels after waking and decreasing around midnight (Pruessner et al., 1997; Bartter et al., 2006). In contrast, chronic insomniacs have significantly higher cortisol levels during the evening (Spath-Schwalbe, 1992).

The hypothalamic nucleus that comprises the HPA is the paraventricular nucleus (PVN) where corticotropin-releasing hormone (CRH) release is key to inducing stress responses and augmenting the levels of ACTH and cortisol. A reciprocal excitatory interaction between the HPA and the orexinergic system has recently been revealed to occur. First, an anatomical interface between these two nuclei has been observed: orexin neurons extensively innervate the PVN, whereas CRH neurons innervate the LH (Winsky-Sommerer et al., 2004). Second, a physiological association has also been reported: there is an enhanced release of CRH that follows the intracerebroventricular (ICV) infusion of orexins (Al-Barazanji et al., 2001; Sakamoto et al., 2004), as well as an activation of orexinergic neurons after CRH administration (Winsky-Sommerer et al., 2004). This anatomical and functional overlap has raised the question of whether or not the orexinergic system is involved in the modulation of stress.

To study the response of orexinergic neurons in stressful situations, experiments have been carried out. In one trial, the activity of orexin-producing neurons in rats was evaluated after they were subjected to a swimming stress test known to increase the amount of ACTH in plasma. During this test the activation of orexinergic cells, measured by c-Fos immunoreactivity, significantly increased, suggesting orexinergic activation associated with stress. Furthermore, the study also showed that pretreatment with an orexin antagonist significantly reduced the amount of ACTH released to plasma (Chang et al., 2007), revealing a role for orexins in this particular stress response. However, it seems that orexin-producing neurons are not activated by all kinds of stress; instead they appear to be specifically recruited by stressful scenarios that require increased attention to environmental cues (Furlong et al., 2009).

The HPA also directly influences the activity of the locus coeruleus (LC), a major source of norepinephrine in the CNS and a very important wake-promoting nucleus (Buckley and Schatzberg, 2005). Orexinergic neurons also have an excitatory influence on the LC, as they activate it during the waking hours of the SWC (Hagan et al., 1999; Bourgin et al., 2000; Del Cid-Pellitero and Garzón, 2011). Although it has not yet been tested, it is possible that repetitive stressful events, requiring attention to environmental cues, activate the HPA and induce the release of CRH, subsequently activating the LC and orexinergic neurons. This would promote attention and inhibit sleep, setting in motion a vicious cycle that could develop into chronic insomnia.

## Rationale for orexin antagonism aimed at the treatment of insomnia

The orexinergic system was first described in the 1990s (de Lecea et al., 1998; Peyron et al., 1998; Sakurai et al., 1998). Shortly thereafter it was linked to the development of the sleep disorder narcolepsy (Chemelli et al., 1999; Lin et al., 1999; Thannickal et al., 2000). Since then, orexins have been intensely studied for their role in the SWC primarily as wake-promoting neurotransmitters (Alexandre et al., 2013).

Orexin producing neurons are found in the LH. These neurons synthesize two excitatory neuropeptides called orexin A and B (OX_A_ and OX_B_, alternatively known as hypocretin 1 and 2) cleaved from a common protein precursor called prepro-orexin (prepro-hypocretin). Orexinergic neurons extensively innervate the CNS (Peyron et al., 1998), specifically areas known for their role in promoting arousal like the LC, TMN, BF, cerebral cortex and dorsal raphe (DR).

Several studies have corroborated the role of the orexinergic system in sustaining wakefulness. For instance, it has been shown that orexinergic neuronal activity is a function of the degree of wakefulness, and is highest during active waking, and decreases during quiet waking and sleep (Kiyashchenko et al., 2002; Lee et al., 2005; Mileykovskiy et al., 2005). In addition, both ICV infusions (Piper et al., 2000; De la Herrán-Arita et al., 2011) and microinjections in sleep control related nuclei (Bourgin et al., 2000; España et al., 2001; Xi et al., 2001) of OX_A_ lengthen the amount of time spent awake in a dose dependent manner. Moreover, the use of optogenetics to activate orexinergic neurons in the LH has been shown to increase the probability of a transition from nREM or REM sleep to waking (Adamantidis et al., 2007).

**Figure 1:**
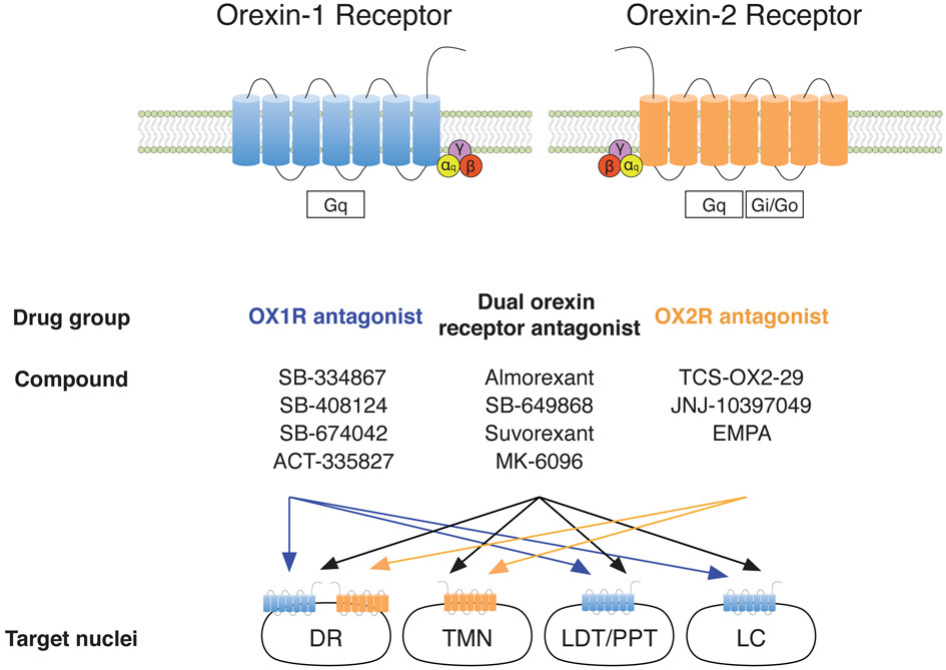
**Orexin receptors and antagonists.** Abbreviations: OX_1_R, type 1 orexin receptor; OX_2_R, type 2 orexin receptor; DR, dorsal raphe; TMN, tuberomammillary nucleus; LDT, laterodorsal tegmental nucleus; PPT, pedunculopontine tegmental nucleus; LC, *locus coeruleus*.

Orexins exert their actions through their interaction with two G protein-coupled receptors called OX_1_R and OX_2_R (hcrt1R and hcrt2R, respectively). These receptors have different affinities for the orexin peptides, while OX_A_ binds to both receptors, OX_B_ selectively binds to OX_2_R (Sakurai et al., 1998) (Figure 1). In addition, orexin receptors are differentially located throughout the CNS; the LC mainly expresses OX_1_R, the TMN and the PVN exclusively express OX_2_R, while the DR, BF and cortex express both receptors (Marcus et al., 2001) (Figure 1).

The distinct distribution and affinities of orexin receptors suggest they play different roles in the maintenance of wakefulness (Table 2). This has been studied using different strains of transgenic mice, such as Knockouts (KO) for either one of the orexin receptors, or both (DKO). These mice show varying degrees of sleep disturbance. While OX_1_R KO mice do not exhibit any obvious behavioral alterations (Sakurai, 2007), OX_2_R KO mice manifest some features of narcolepsy, including an inability to sustain wakefulness (Willie et al., 2003). DKO mice display the most profoundly disturbed sleep phenotype of all three models: narcolepsy with cataplexy (transient episodes of behavioral arrest) (Kalogiannis et al., 2011). The robust narcoleptic phenotype in DKO mice indicates a synergistic role between OX_1_R and OX_2_R in the maintenance of wakefulness.

**Table 2:** **Summary of orexin receptor antagonists**.

**Name**	**Affinity (K_i_, nM)**			**Possible applications**
	**OX_1_R**	**OX_2_R**		
**SINGLE OREXIN**			**SELECTIVITY**	
**RECEPTOR ANTAGONIST**				
SB-334867	28	1704	OX_1_R	Withdrawal, substance abuse, obesity, panic disorder
SB-408124	22	1405		
SB-674042	1.1	129		
ACT-335827	6	417 (IC_50_)	
TCS-OX2-29	–	7.4 (pKi)	OX_2_R	Sleep promotion
JNJ-10397049	1644	6		
EMPA	900	1.1		
Antagonist 26	6.34	7.23 (pKi)	
**DUAL OREXIN**			**FDA PHASE**	
**RECEPTOR ANTAGONIST**				
Almorexant	13	8	III (discontinued)	Treatment of insomnia	
SB-649868	0.3	0.4	II (completed)
Suvorexant	0.6	0.4	III (pending approval)
MK-6096	2.5	0.3	–	–
DORA 30	18	7 (IC_50_)	–	Sleep promotion

To further characterize the role of orexin receptors, selective orexin receptor KO mice were stimulated with ICV infusions of OX_A_. Specific stimulation of OX_1_R in OX_2_R KO mice produced a moderate improvement in wakefulness and suppression of nREM, whereas the stimulation of OX_2_R in OX_1_R KO mice resulted in a greatly enhanced wakefulness (Mieda et al., 2011). This suggests that OX_1_R plays an important role in suppressing the instigation of nREM sleep, while OX_2_R has a major role in promoting wakefulness.

In another direction, overexpression of components of the orexinergic system also disrupts the SWC. For example in the zebrafish, overexpression of orexinergic neurons has been shown to induce an insomnia-like phenotype (Prober et al., 2006). Mice that overexpress prepro-orexin display sleep abnormalities which include fragmentation of nREM sleep, reduced REM sleep, and increased motor activity during REM sleep, suggesting an inability to maintain sleep states (Willie et al., 2011).

If we take into consideration that the activation of the orexinergic system promotes wakefulness and that its disruption brings about sleep disturbances, orexin antagonists could offer a very effective therapeutic alternative for insomnia.

## Orexin antagonists for treating insomnia

The newest molecules in the pipeline for the treatment of insomnia are orexin antagonists. There are many orexin antagonists currently being studied for the treatment of insomnia and they fall into one of two categories: single orexin receptor antagonists (SORAs) and dual orexin receptor antagonists (DORAs).

In the following part of this review, we evaluate the effectiveness of these drugs for the treatment of insomnia. A summary of orexin antagonists is provided in Table 2.

### SORAs

Evidence from experiments conducted in transgenic models of orexin receptor KO mice suggests that SORAs targeting OX_1_R will not promote sleep as effectively as those aimed at OX_2_R.

#### OX_1_R

Of the available SORAs, SB-334867 was the first drug designed to selectively antagonize OX_1_R (Smart et al., 2001). This SORA is able to counteract the suppression of REM sleep after ICV infusion of OX_A_ in rats. However, it does not decrease wakefulness, or increase the amount of time spent in sleep, nor does it reduce sleep latency by itself at any given dose (Smith et al., 2003). Morairty and colleagues, later noted that SB-334867 at 3 and 30 mg/kg increased cumulative nREM during the first 4 and 6 h following administration (Morairty et al., 2012). SB-334867 is classified as a selective OX_1_R antagonist, but unspecific binding to adenosine and serotonin receptors has been reported; it also affects monoamine and norepinephrine transporters at high concentrations (Lebold et al., 2013).

Although the effect of SB-334867 on sleep induction was poor, this molecule has proven to be useful for the treatment of other conditions, such as substance abuse, withdrawal, obesity and panic disorder (White et al., 2005; Johnson et al., 2010; Jupp et al., 2011; Smith and Aston-Jones, 2012).

Other selective OX_1_R antagonists include SB-408124, SB-674042 and the newest AK-335827. So far, neither SB-408124 nor AK-335827 have been found to promote sleep (Dugovic et al., 2009; Steiner et al., 2013). In the case of SB-408124 however, insufficient brain penetration was found and this could account in part for the absence of observable effects (Morairty et al., 2012).

There are few studies characterizing the effect of these antagonists; nonetheless, there is some evidence that they can be useful in the treatment of substance abuse and withdrawal, and have potential for treating obesity and panic disorder. For example, it has been shown that subcutaneous administration of SB-408124 lowers the release of dopamine in the *nucleus accumbens* (Dugovic et al., 2009), and orally administered AK-335827 has anxiolytic effects (Steiner et al., 2013).

It is interesting that despite the lack of sleep-promoting effects of OX_1_R SORAs on their own, these compounds have the capacity to thwart the sleep inhibiting effects of ICV orexin infusion (Smith et al., 2003). Strikingly, they can also reduce the sleep-promoting effects of other antagonists; as observed under the coadministration of OX_1_R and OX_2_R antagonists which has a milder sleep-promoting effect than when the OX_2_R antagonist is administered by itself (Dugovic et al., 2009). This could be due to the high concentrations used in these experiments (30 mg/kg) and the unspecific binding that follows.

#### OX_2_R

Type 2 orexin receptors are selectively expressed both in the PVN and the TMN. As mentioned above, the PVN is part of the HPA, and the overactivation of the HPA has been proposed to be involved in the etiology of PI. Withholding the orexinergic stimuli to the HPA could help prevent the development of the vicious cycle proposed earlier. Additionally, the TMN, a histaminergic nucleus, has a major role in the arousal effect observed after orexinergic stimulation (Huang et al., 2001). Inhibition of the TMN with orexinergic antagonists could, facilitate the induction of sleep by allowing the sleep promoting nuclei to prevail.

OX_2_R antagonists are less common than the other classes. Among the few available molecules that have been studied in the context of sleep promotion are EMPA, TCS-OX2-29 and JNJ-10397049. These antagonists have been more successful at diminishing wakefulness than OX_1_R antagonists.

EMPA is the least effective sleep-promoting OX_2_R SORA studied. While intraperitoneal administration of EMPA (100 mg/kg) has been shown to selectively increase cumulative nREM sleep during the first 4 and 6 h after administration, these increases are not accompanied by any significant increase in REM sleep or reduction in latencies for either sleep stage (Morairty et al., 2012). On the other hand, rats that received an ICV infusion of TCS-OX2-29 (40 nmol) increased their total sleep time by 7% in comparison to controls that received saline infusions. Interestingly, this effect was secondary to a selective increase in REM sleep (Kummangal et al., 2013).

Intraperitoneal administration (5, 25 or 50 mg/kg) of JNJ-10397049 6 h into the dark phase, produced a robust increase in total sleep time, traced to increases in both REM and nREM sleep (Gozzi et al., 2011). Similar results have been observed with subcutaneous injections (Dugovic et al., 2009). Starting at doses of 3 mg/kg, administration of JNJ-10397049 2 h into the light phase significantly decreased the latency to nREM sleep while increasing the length of each bout. At higher concentrations (30 mg/kg), this drug also induced a decrease in REM sleep latency without noticeable changes in its duration. Overall, 3 mg/kg of JNJ-10397049 increased total sleep time by 42% while keeping the proportion of nREM/REM sleep observed in vehicle treated animals.

Furthermore, microdialysis assays showed that this compound reduces histamine release in the LH (Dugovic et al., 2009). As mentioned earlier, release of histamine in the TMN is fundamental for the wake-promoting effects of OX_A_ ICV infusions (Huang et al., 2001).

Animal studies support the notion that OX_2_R antagonists are helpful as sleep inducing agents. Further research is needed to determine the degree of sleep generation achieved by these compounds in different species, including humans. It is possible that the sleep-promoting effect of selectively antagonizing OX_2_R is less pronounced than the one observed with DORAs, but it may also be more specific, which would be worth investigating.

#### DORAs

It had been long suspected that antagonizing both orexin receptors would elicit the most powerful sleep-promoting effects; therefore, many of the studies around orexin antagonists have focused on DORAs. So far, evidence has proven this to be the case (Morairty et al., 2012), to the point that DORAs are the only orexin antagonists currently undergoing clinical trials in the hope that they will be approved by the FDA for the treatment of insomnia.

#### Almorexant

ACT-078573 (almorexant) is the most widely studied DORA and one of the first to enter phase III clinical trials (NCT00608985).

In wild type mice, the administration of almorexant 15 min before lights-out reduced the amount of time spent awake, while increasing the length of nREM and REM sleep bouts in a dose dependent manner (Mang et al., 2012). Notably, the proportion of REM sleep observed after almorexant administration during the dark phase was in the range of that observed during the light phase with vehicle treatment.

Further studies in KO mice determined that the sleep-inducing effect of almorexant was related to the stimulation of OX_2_R and not OX_1_R. This conclusion was reached after the authors did not observe any changes in the amount of sleep in OX_2_R KO, but did for OX_1_R KO mice. Interaction with sites other than orexin receptors that could account for the changes in sleep times was discarded when no changes were observed in the SWC of DKO mice.

When administered in healthy humans, almorexant was well tolerated. Doses of and above 200 mg elicited decreased alertness, with increased reports of fatigue, drowsiness, sleepiness, and sleep efficiency, measured as an increase in SWS and REM sleep (Brisbare-Roch et al., 2007). In PI patients, it proved to be effective for boosting sleep, increasing total sleep time, and reducing both REM sleep latency and the frequency of awakening (Hoever et al., 2012). This effect was dose dependent, with the most notorious effect on sleep architecture achieved at doses of 400 mg; doses of 100 and 200 mg had modest effects on sleep, with fewer adverse effects (e.g., headache, dizziness, blurred vision).

Although almorexant appeared to be well tolerated, the pharmaceutical companies sponsoring this drug discontinued the clinical trials in 2011 citing “safety observations” that required further evaluation. Currently, almorexant is in a new phase of clinical trials in order to evaluate its effect on cognitive performance (NCT01243060).

#### SB-649868

SB-649868 is a potent orally active DORA manufactured by the same pharmaceutical company as almorexant. There is also evidence for the effectiveness of SB-649868 in promoting sleep, both in animal studies and human trials.

When dispensed to rats, it elicited an increase in total sleep time (related to increases of both nREM and REM sleep) and reduced sleep latencies at doses of 10 and 30 mg. Moreover, the effect of SB-649868 on motor coordination was null, given that the rotarod model of coordination failed to reveal any motor impairment in rats treated with this compound, even when the orexin antagonist was administered concurrently with ethanol (Di Fabio et al., 2011). Compared to almorexant, the *in vivo* efficacy of this compound is excellent, thus it has been moved on to clinical trials.

The administration of SB-649868 to healthy volunteers who participated in a noise-disturbed sleep study showed that this compound is effective at inducing somnolence and fatigue at 10 and 30 mg doses (Bettica et al., 2012). Furthermore, patients diagnosed with PI reported that SB-649868 significantly improved the quality of sleep (10, 30, and 60 mg) while objectively increasing total sleep time, reducing sleep latency and suppressing nighttime awakenings (Bettica et al., 2012). During this study, the most common complaints were headaches, dry mouth and nasopharyngitis; the number of complaints increased in a dose dependent manner. Phase II clinical trials of SB-649868 have been completed (NCT00426816).

#### Suvorexant

Another promising DORA is the potent MK-4305 (suvorexant), a compound variation from the diazepane series. Animal studies have shown that suvorexant reduces active wake time by increasing nREM and REM sleep in rats, dogs, and monkeys (Winrow et al., 2011). In all cases, these effects were achieved at much lower doses (10 mg) than with almorexant.

This molecule is also in phase III clinical trials (NCT01097616) and is currently under evaluation for approval by the FDA. In healthy humans, the lowest dose (10 mg) reduced the number of awakenings after sleep onset; and at higher doses (50 mg) it reduced sleep latency, while increasing sleep efficiency and total sleep time (Sun et al., 2013). High doses (50 and 100 mg) elicit undesirable side effects such as an increase in reaction time, difficulty waking up and reduced alertness following awakening; in addition it leads to mild complaints of headaches and somnolence.

When administered to PI patients, suvorexant reduced sleep latency and increased the time patients spent asleep after a single administration without reducing the number of awakenings after sleep onset. The increase in total sleep time was mostly attributable to an increase in REM sleep. The most frequent adverse effects were somnolence, headaches, dizziness and abnormal dreams, all of which occurred in a dose dependent manner. In addition, there were no next-day residual effects, no rebound insomnia, complex sleep-related behaviors or withdrawal effects after 4 weeks. Instead, during this study there were a few reports of sleep paralysis (1, *n* = 59, at 40 mg), and at high doses (80 mg), excessive daytime sleepiness (1, *n* = 61), and hypnagogic hallucinations (1, *n* = 61) (Herring et al., 2012). These are symptoms of narcolepsy, and should be carefully monitored due to the close association between narcolepsy and the orexinergic system.

In general, suvorexant was well tolerated and, because the most consistently effective dosages were 30 and 40 mg, the pharmaceutical company manufacturing suvorexant submitted a dose range of 15–40 mg for FDA approval. To date, suvorexant has not been approved and the FDA has requested a lower starting dose of 10 mg for the general population and a 5 mg dose for those taking concomitant CYP3A4 inhibitors.

One potential advantage of DORAs over classic insomnia treatments, such as BZDs, is the possibility of inducing a more physiological sleep. For instance, while DORAs enhance REM sleep, BZDs have proven to suppress this sleep stage (Lanoir and Killam, 1968; Borbély et al., 1985; Gaillard et al., 2009). In addition, orexin antagonists appear to have a better side effect profile, with mild complaints of headaches and dizziness being the most common. The only exception appears to be almorexant, given the surprising suspension of clinical trials. Although the reasons for halting clinical trials have not been disclosed to the public, it is conceivable that the high doses required to achieve therapeutic effects could also cause more severe adverse effects, not observed in other drugs that require doses 10 times smaller.

One of the most important questions when characterizing an orexin antagonist is whether or not it elicits narcoleptic symptoms. Thus far, orexin antagonists have not been observed to cause cataplexy in animal models or in human patients. Up until now, reports of human patients complaining of sleep paralysis or hypnagogic hallucinations have been scarce, only occurring with high doses of suvorexant. As clinical trials progress, medical practitioners should still be on the alert for symptoms of narcolepsy.

## Discussion

The research and evaluation of new insomnia treatments is often complex, given that insomnia is usually of multifactorial etiology. Understanding the molecular and receptor mechanisms involved in promoting sleep in a variety of disorders could provide future approaches to new drug development.

An abundance of current research data has demonstrated the importance of the orexin system in the regulation of the SWC. Excitement over the potential of orexin receptor antagonists for treating insomnia peaked in 2007 when Actelion Pharmaceuticals Ltd. revealed that almorexant significantly decreased wakefulness in rats, dogs and humans, without evidence of cataplexy; unfortunately, clinical trials were discontinued in 2011 due to safety concerns that required further evaluation. Second generation inhibitors with improved pharmaceutical properties are currently being developed and tested for the potential treatment of insomnia and other disorders linked to dysfunction in the orexin system.

Orexin antagonism may offer improved avenues for combining medications with non-drug treatments such as CBTi for insomnia. However, more randomized controlled trials are needed to assess both the short- and long-term effects of these medications, as well as their efficacy in comorbid diseases that affect the quality and quantity of sleep.

### Conflict of interest statement

The authors declare that the research was conducted in the absence of any commercial or financial relationships that could be construed as a potential conflict of interest.
